# PangenePro: an automated pipeline for rapid identification and classification of gene family members

**DOI:** 10.1093/bioadv/vbaf159

**Published:** 2025-07-02

**Authors:** Kinza Fatima, Haifei Hu, Muhammad Tahir ul Qamar

**Affiliations:** Integrative Omics and Molecular Modeling Laboratory, Department of Bioinformatics and Biotechnology, Government College University Faisalabad (GCUF), Faisalabad 38000, Pakistan; College of Natural & Agricultural Sciences, University of California, Riverside, CA 92521, United States; Rice Research Institute, Guangdong Academy of Agricultural Sciences & Key Laboratory of Genetics and Breeding of High Quality Rice in Southern China (Co-construction by Ministry and Province), Ministry of Agriculture and Rural Affairs & Guangdong Key Laboratory of Rice Science and Technology, Guangzhou 510640, China; Integrative Omics and Molecular Modeling Laboratory, Department of Bioinformatics and Biotechnology, Government College University Faisalabad (GCUF), Faisalabad 38000, Pakistan

## Abstract

**Motivation:**

The increasing availability of sequenced and assembled plant genomes in public databases has led to a surge in genome-wide identification (GWI) studies of gene families. However, previous studies are often single-reference genome-based, limiting their ability to capture intraspecific genetic diversity. Further, manual identification from multiple genomes is labor-intensive and time-consuming.

**Results:**

Here, we present PangenePro, a fully automated pipeline using Python and R scripting, implemented in the Linux environment, designed to identify and classify gene family members across multiple genomes simultaneously. This pipeline integrates sequence alignment using BLAST, domain validation through InterProScan, and orthologous clustering to classify the identified genes into core, dispensable, and unique pangenes sets. PangenePro was tested using five Arabidopsis thaliana, three Arachis and rice, and five Barley genomes, identifying a number of members comparable to those in previously reported studies. These results demonstrate the accuracy and efficiency of this method for gene family identification and classification in diverse and complex genomes. Moreover, its rapid nature enables comprehensive capture of intraspecific diversity and yields valuable candidate genes for further functional and plant breeding studies.

**Availability and implementation:**

The PangenePro is freely available at GitHub DOI: https://github.com/kinza111/PangenePro.

## 1 Introduction

For the last two decades, advances in genomics technologies, particularly the high-throughput next-generation sequencing, have resulted in the generation as well as storage of enormous amounts of omics datasets in publicly available databases (Liu *et al.* 2022). These datasets have enabled various gene family identification and analysis studies using GWI approaches, which apply various integrated omics and bioinformatics tools and resources to explore gene families and their structural and functional diversity. These *in silico* analyses provide an effective way to understand the evolutionary relationships among the members of a gene family (Fatima *et al.* 2023). However, most previous studies using this approach have been based on a single reference genome. Recent genome sequencing and comparative studies have shown that a single reference genome does not represent the entire genetic makeup of multiple individuals belonging to the same species, leading to imprecise estimation of genetic diversity (Giordano *et al.* 2018, Tahir Ul Qamar *et al.* 2019, Hu *et al.* 2024). This has led to the adoption of the pangenome concept, which emphasizes the analysis of multiple genomes rather than individual reference genomes (Tahir ul Qamar *et al.* 2020, Hu *et al.* 2025). In this context, a pangene refers to an allele or a gene model shared by some or all members of a species at a particular genomic region (Moreira *et al.* 2023). Few tools have been developed to identify the Presence-Absence variations (PAV) regions at the genome level. ScanPAV performs a pairwise comparison of genome assemblies to extract PAVs (Giordano *et al.* 2018). Another tool called get_pangenes also requires pairwise whole-genome alignment to identify pangenes in a set of genomes (Moreira *et al.* 2023).

In this note, we present a new pipeline, PangenePro, that utilizes available annotated genomes to identify members of a specific gene family of interest. The pipeline also delivers comprehensively classified pangene sets identified across multiple genomes.

## 2 Methods

**Figure 1.**
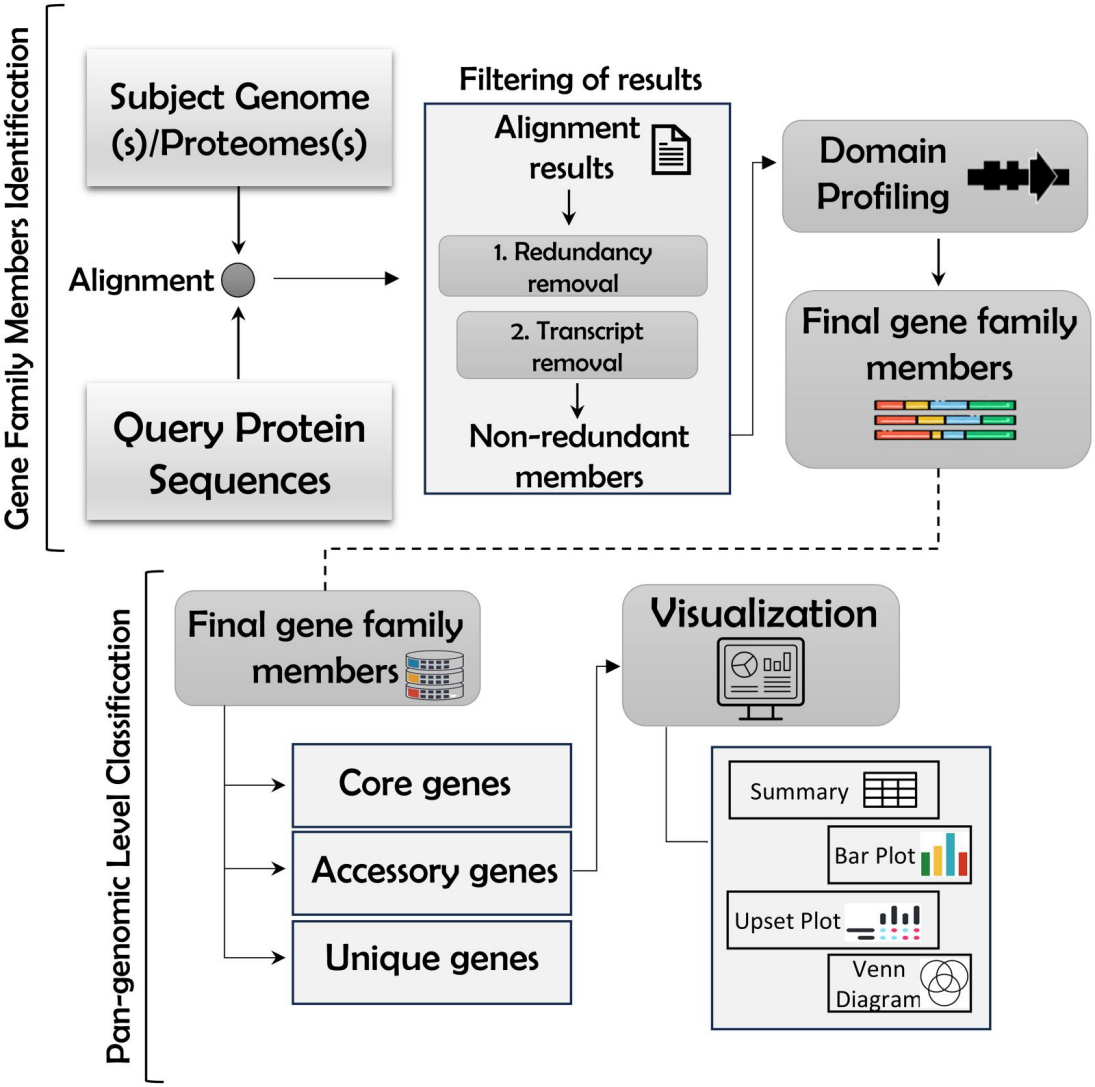
Flowchart showing the fundamental algorithmic steps of the PangenePro pipeline.

An overview of the key algorithmic steps of the PangenePro pipeline is shown in Fig. 1. This framework accepts genome, proteome, and annotation files of the subject genomes, along with a protein FASTA file containing the reference gene family sequences, as input. It first performs the identification of gene family from the queried genomes, followed by classifying these members into pangene sets through the following step: (i) The query protein sequences (representing the gene family of interest, previously identified in model species) are aligned with the input reference proteome files using BLASTp (Camacho *et al.* 2009); (ii) the output gene family accessions are subjected to redundancy and isoform removal. The seqtk (https://github.com/lh3/seqtk) (Li 2012) tool is used to process the protein fasta and annotation files for isoform removal; (iii) The filtered hits are then subjected to domain profiling to validate the final gene family members. The domain profiling is performed using the InterProScan REST API (Blum *et al.* 2021). This step validates the final gene family member accessions as well as their protein fasta files; (iv) These protein fasta sequences identified across multiple genomes are used for orthologous clustering to classify pangenes using OrthoVenn (Xu *et al.* 2019), which used Diamond (Buchfink *et al.* 2015) for aligning the protein fasta sequences; (v) The orthologous-based gene family member clusters are parsed into pangene sets. MCL (Enright *et al.* 2002) and orthAgogue (Ekseth *et al.* 2014) are used to generate clusters of gene family members that are further classified into pangene sets. The clusters containing orthologs from all input genomes are classified as “core” clusters, those with orthologs from more than one input genome are classified as “dispensable” clusters, and the clusters with members from only a single genome are classified as “unique” clusters; (vi) Finally, the output is presented as a summary table that includes pangene clusters, Venn diagrams, the Upset plots, and bar plots, providing a comprehensive visualization of the results.

The scripts of the PangenePro pipeline are written in Python, R, and implemented in Bash. To execute this Linux-based pipeline, users need to pre-install a few necessary tools. The details of these dependencies are provided in the Note 1, available as supplementary data at *Bioinformatics Advances* online. We tested PangenePro on both large and small plant genomes to benchmark its performance against previously reported gene family members. 44 Cysteine-rich receptor-like kinases (*CRK*) genes previously identified in *Arabidopsis thaliana* genome (Wrzaczek *et al.* 2010) were used as queries to identify these gene family members in two simple and model plant genomes, *Arabidopsis* and rice, as well as two complex plants genomes, *Arachis* and Barley. The genomes analyzed included five *Arabidopsis* accessions (Bur_0.v7, Can_0.v7, Ct_1.v7, Edi_0.v7, and Col_0; Gan *et al.* 2011), three rice cultivars (*Oryza sativa* Nipponbare, *O.sativa* indica 93–11, and *O.sativa* Zhenshan 97; Moreira *et al.* 2023), three Arachis species (*A.hypogaea*, *A.duranensis*, and *A.ipaensis*), and five barley genomes (MorexV2, Akashinriki, Barke, Hockett, and Igri; Moreira *et al.* 2023) (Table 1, available as supplementary data at *Bioinformatics Advances* online).

## 3 Results

PangenePro pipeline accepts genome and proteome files in fasta format with extensions such as *.fa, *.faa, *.fasta. Annotation files are accepted in formats with extensions *gff, *.gff3 eand *.gtf. We applied this pipeline to identify members of the CRK gene family across genomes of varying sizes and complexity. In the small diploid genomes of five *A.thaliana* ecotypes, PangenePro identified 42, 40, 42, 42, and 41 *CRK* members in *Bur_0.v7*, *Can_0.v7*, *Ct_1.v7*, *Edi_0.v7*, *Col_0*, respectively (Fig. 1, available as supplementary data at *Bioinformatics Advances* online). These results are consistent with previous reports of 44 CRKs in *Arabidopsis* and validate the pipeline’s specificity. Ortholog clustering across the five ecotypes yielded 28, 24, 27, 26, and 29 clusters, which were subsequently classified into 38 core, 21 dispensable, and 5 unique clusters. In the larger and more complex *Arachis* genomes, PangenePro identified 498, 378, and 332 CRK genes in *A.hypogaea*, *A.duranensis*, and *A.ipaensis*, respectively (Fig. 2, available as supplementary data at *Bioinformatics Advances* online), which notably exceeds the previously reported numbers (Fatima *et al.* 2023). These increases reflect this pipeline sensitivity and ability to identify divergent members even in highly diverse and complex genomes. Clustering in *Arachis* yielded 148, 208, and 167 orthologous clusters, with 72 core clusters shared across all three species, and dispensable clusters shared between genome pairs ranging from 17–75. Unique clusters numbered 18 (*A.hypogaea*), 20 (*A.duranensis*), and 3 (*A.ipaensis*). To further validate its performance, PangenePro was also tested in rice and barley. In three *Oryza sativa* genomes (Japonica Nipponbare, Indica 93–11, and Zhenshan 97), 201, 189, and 176 CRKs were identified, respectively. Orthologous clustering yielded 37–45 clusters across these rice genomes, including 59 core clusters present in all three genomes. Dispensable clusters shared between two genomes were 23 (Nipponbare and indica), 28 (Zhenshan and indica), and 71 (Nipponbare and Zhenshan), while unique clusters were 23, 25, and 20 (Fig. 3, available as supplementary data at *Bioinformatics Advances* online). In five barley cultivars (MorexV2, Akashinriki, Barke, Hockett, and Igri), 76, 68, 63, 72, and 73 CRK genes were identified, respectively. Orthologous clustering across these genomes produced 31 core clusters shared by all cultivars. Dispensable clusters shared between subsets of genomes ranged from 2 to 19 clusters, while unique clusters numbered 2 in MorexV2, 3 in Akashinriki, 3 in Barke, 4 in Hockett, and 2 in Igri (Fig. 4, available as supplementary data at *Bioinformatics Advances* online). These results highlight the variability in CRK family size and composition among barley cultivars. The higher number of CRKs identified in rice compared with the 36 reported by Shumayla *et al.* (2019) reflects both the optimized similarity thresholds implemented in PangenePro and the exceptional annotation quality of the rice reference genomes. As a well‐studied model species, rice benefits from comprehensive assembly and annotation, yielding more predicted members than barley, whose genome resources remain comparatively less complete. These results underscore PangenePro’s robustness in detecting homologous gene family members across both closely related and highly diverse genomes, while also highlighting that performance is contingent upon the quality of the input assembly and annotation. Further, an extensive benchmarking analysis comparing PangenePro with 12 other relevant pipelines and tools also highlights the core methodology, approach, and how our pipeline is unique compared to those tools/resources (Table 2, available as supplementary data at *Bioinformatics Advances* online). Overall, PangenePro delivers accurate, efficient, and rapid comparative gene family analysis at the pangenome level, capable of processing multiple genomes simultaneously.

## 4 Conclusion

Plant gene family members play an important role in stress regulation and exhibit variations across different genomes, necessitating multi-genome analyses to capture their full diversity. Here, we introduce PangenePro, an automated pipeline for the comprehensive identification and classification of gene family members across multiple genomes. By integrating sequence similarity searches, domain validation, and ortholog clustering, PangenePro reliably partitions gene sets into core, dispensable, and unique categories across genomes of varying complexity. We validated the pipeline on *Arabidopsis*, *Arachis*, rice, and barley genomes, which demonstrated the pipleine’s high efficiency and robustness. PangenePro provides a novel framework for exploring gene-level variation and prioritizing candidate genes for functional genomics and crop improvement studies.

## Supplementary Material

vbaf159_Supplementary_Data

## Data Availability

The source code and installation instructions for this pipeline are available at https://github.com/kinza111/PangenePro and can be accessed directly from source from the PangenePro GitHub repository.

## References

[vbaf159-B1] Blum M , Chang H-Y, Chuguransky S et al The InterPro protein families and domains database: 20 years on. Nucleic Acids Res 2021;49:D344–54. 10.1093/nar/gkaa977 33156333 PMC7778928

[vbaf159-B2] Buchfink B , Xie C, Huson DH. Fast and sensitive protein alignment using DIAMOND. Nat Methods 2015;12:59–60. 10.1038/nmeth.3176 25402007

[vbaf159-B3] Camacho C , Coulouris G, Avagyan V et al BLAST+: architecture and applications. *BMC Bioinform* 2009;**9**:1–9. 10.1186/1471-2105-10-421 PMC2803857 20003500

[vbaf159-B4] Ekseth OK , Kuiper M, Mironov V. orthAgogue: an agile tool for the rapid prediction of orthology relations. Bioinformatics 2014;30:734–6. 10.1093/bioinformatics/btt582 24115168

[vbaf159-B5] Enright AJ , Van Dongen S, Ouzounis CA. An efficient algorithm for large-scale detection of protein families. Nucleic Acids Res 2002;30:1575–84. 10.1093/nar/30.7.1575 11917018 PMC101833

[vbaf159-B6] Fatima K , Sadaqat M, Azeem F et al Integrated omics and machine learning-assisted profiling of cysteine-rich-receptor-like kinases from three peanut spp. revealed their role in multiple stresses. Front Genet 2023;14:1252020. https://www.frontiersin.org/articles/10.3389/fgene.2023.1252020 37799143 10.3389/fgene.2023.1252020 PMC10547876

[vbaf159-B7070] Gan X, Stegle O, Behr J et al Multiple reference genomes and transcriptomes for Arabidopsis thaliana. Nature 2011;477:419–23. 21874022 10.1038/nature10414 PMC4856438

[vbaf159-B7] Giordano F , Stammnitz MR, Murchison EP et al scanPAV: a pipeline for extracting presence–absence variations in genome pairs. Bioinformatics 2018;34:3022–4. 29608694 10.1093/bioinformatics/bty189 PMC6129304

[vbaf159-B8] Hu H , Wang J, Nie S et al Plant pangenomics, current practice and future direction. Agriculture Commun 2024;2:100039. 10.1016/j.agrcom.2024.100039

[vbaf159-B9] Hu H , Zhao J, Thomas WJW et al The role of pangenomics in orphan crop improvement. Nat Commun 2025;16:118. 10.1038/s41467-024-55260-4 39746989 PMC11696220

[vbaf159-B10] Li H. seqtk toolkit for processing sequences in FASTA/Q formats. GitHub 2012;767:69.

[vbaf159-B11] Liu H , Wang X, Liu S et al Citrus pan-genome to breeding database (CPBD): a comprehensive genome database for citrus breeding. Mol Plant 2022;15:1503–5. 10.1016/j.molp.2022.08.006 36004795

[vbaf159-B12] Moreira BC , Saraf S, Naamati G et al GET _ PANGENES : calling pangenes from plant genome alignments confirms presence-absence variation. Genome Biol 2023;24:223. 10.1186/s13059-023-03071-z 37798615 PMC10552430

[vbaf159-B7000] Shumayla, Tyagi S, Sharma A et al Genomic dissection and transcriptional profiling of Cysteine-rich receptor-like kinases in five cereals and functional characterization of TaCRK68-A. Int J Biol Macromol 2019;134:316–29. 31078592 10.1016/j.ijbiomac.2019.05.016

[vbaf159-B13] Tahir Ul Qamar M , Zhu X, Khan MS et al Pan-genome: a promising resource for noncoding RNA discovery in plants. Plant Genome 2020;13:e20046. 10.1002/tpg2.20046 33217199 PMC12806977

[vbaf159-B14] Tahir Ul Qamar M , Zhu X, Xing F et al ppsPCP: a plant presence/absence variants scanner and pan-genome construction pipeline. Bioinformatics 2019;35:4156–8. 10.1093/bioinformatics/btz168 30851098

[vbaf159-B15] Wrzaczek M , Brosché M, Salojärvi J et al Transcriptional regulation of the CRK/DUF26 group of Receptor-like protein kinases by ozone and plant hormones in Arabidopsis. *BMC Plant Biol* 2010;1:1–19. 10.1186/1471-2229-10-95 PMC3095361 20500828

[vbaf159-B16] Xu L , Dong Z, Fang L et al OrthoVenn2: a web server for whole-genome comparison and annotation of orthologous clusters across multiple species. Nucleic Acids Res 2019;47:W52–8. 10.1093/nar/gkz333 31053848 PMC6602458

