# Supplementary material for: PangenePro: an automated pipeline for rapid identification and classification of gene family members
Source: Bioinform Adv. 2025 Jul 2;5(1):vbaf159. doi: 10.1093/bioadv/vbaf159 (PMC12255874; doi:10.1093/bioadv/vbaf159)
Supplement: vbaf159_Supplementary_Data [file vbaf159_supplementary_data.docx]

Genome Analysis

**PangenePro: an automated pipeline for rapid identification and classification of gene family members**

Kinza Fatima^1,2^, Haifei Hu^3^, Muhammad Tahir ul Qamar^1,*^

^1^Integrative Omics and Molecular Modeling Laboratory, Department of Bioinformatics and Biotechnology, Government College University Faisalabad (GCUF), Faisalabad, 38000, Pakistan; [tahirulqamar@gcuf.edu.pk](mailto:tahirulqamar@gcuf.edu.pk)

^2^College of Natural & Agricultural Sciences, University of California, Riverside, CA 92521, USA; [kfati002@ucr.edu](mailto:kfati002@ucr.edu)

^3^Rice Research Institute, Guangdong Academy of Agricultural Sciences & Key Laboratory of Genetics and Breeding of High Quality Rice in Southern China (Co-construction by Ministry and Province), Ministry of Agriculture and Rural Affairs & Guangdong Key Laboratory of Rice Science and Technology, Guangzhou, 510640, China; [huhaifei@gdaas.cn](mailto:huhaifei@gdaas.cn)

*Corresponding author: [tahirulqamar@gcuf.edu.pk](mailto:tahirulqamar@gcuf.edu.pk)

Associate Editor: Michael DeGiorgio

**Supplementary note 1: List of dependencies for PangenePro.**

PangenePro supports **Linux** systems due to the following software dependencies which need to be pre-installed on the user’s system and require Python 3.8 or higher version, and R.

| Blast+ | [wget https://ftp.ncbi.nlm.nih.gov/blast/executables/blast+/LATEST/ncbi-blast-2.16.0+-x64-linux.tar.gz](wget%20https:/ftp.ncbi.nlm.nih.gov/blast/executables/blast+/LATEST/ncbi-blast-2.16.0+-x64-linux.tar.gz) |
| --- | --- |
| Seqtk | <https://github.com/lh3/seqtk> |
| Diamond | <https://github.com/bbuchfink/diamond> |
| orthAgogue | <https://github.com/samyeaman/orthagogue> |
| MCL | <https://github.com/micans/mcl> |

## **Running the Application**

Clone the repository:

*git clone https://github.com/yourusername/PangenePro.git
cd PangenePro*

Set up your environment and install dependencies. The query protein sequence, proteome, and genomes files must be in fasta format (.fasta, .faa, .fa) format. The subject genome must be assembled at the chromosomal level. The annotation file must be in .gff, .gff3, or .gtf format.

Make suggested changes in *PangenePro.sh* script and run the following command to execute the pipeline by setting query and subject files in the *PangenePro.sh* script and running it:

*$ bash PangenePro.sh*

**Supplementary Table 1:** Summary of the dataset used as a test case to benchmark the PangenePro pipeline. The size of genomes, proteomes, and annotation files of three *Arachis* species and five *Arabidopsis* ecotypes (Mbs/M and Gbs/G).

| **Species genome** | **Genome** | **Proteome** | **Annotation** |
| --- | --- | --- | --- |
| ***Arabidopsis*** | | | |
| Bur_0.v7 | 115M | 16M | 51M |
| Can_0.v7 | 114M | 16M | 51M |
| Ct_1.v7 | 115M | 16M | 50M |
| Edi_0.v7 | 115M | 16M | 51M |
| Col_0 | 116M | 15M | 51M |
| ***Arachis*** | | | |
| *A. hypogaea* | 2.5G | 52M | 455M |
| *A. duranensis* | 1.1G | 23M | 189M |
| *A. ipaensis* | 1.3G | 28M | 256M |
| **Rice** | | | |
| *O. sativa* Japonica Group Nipponbare | 373M | 22M | 202M |
| *O. sativa* indica 93–11 | 414M | 24M | 213M |
| *O. sativa* Zhenshan 97 | 327M | 19M | 187M |
| **Barley** | | | |
| MorexV2 | 4,210M | 22M | 76M |
| Akashinriki | 4,401M | 22M | 77M |
| Barke | 4,073M | 20M | 67M |
| Hockett | 4,201M | 22M | 74M |
| Igri | 4,202M | 22M | 74M |


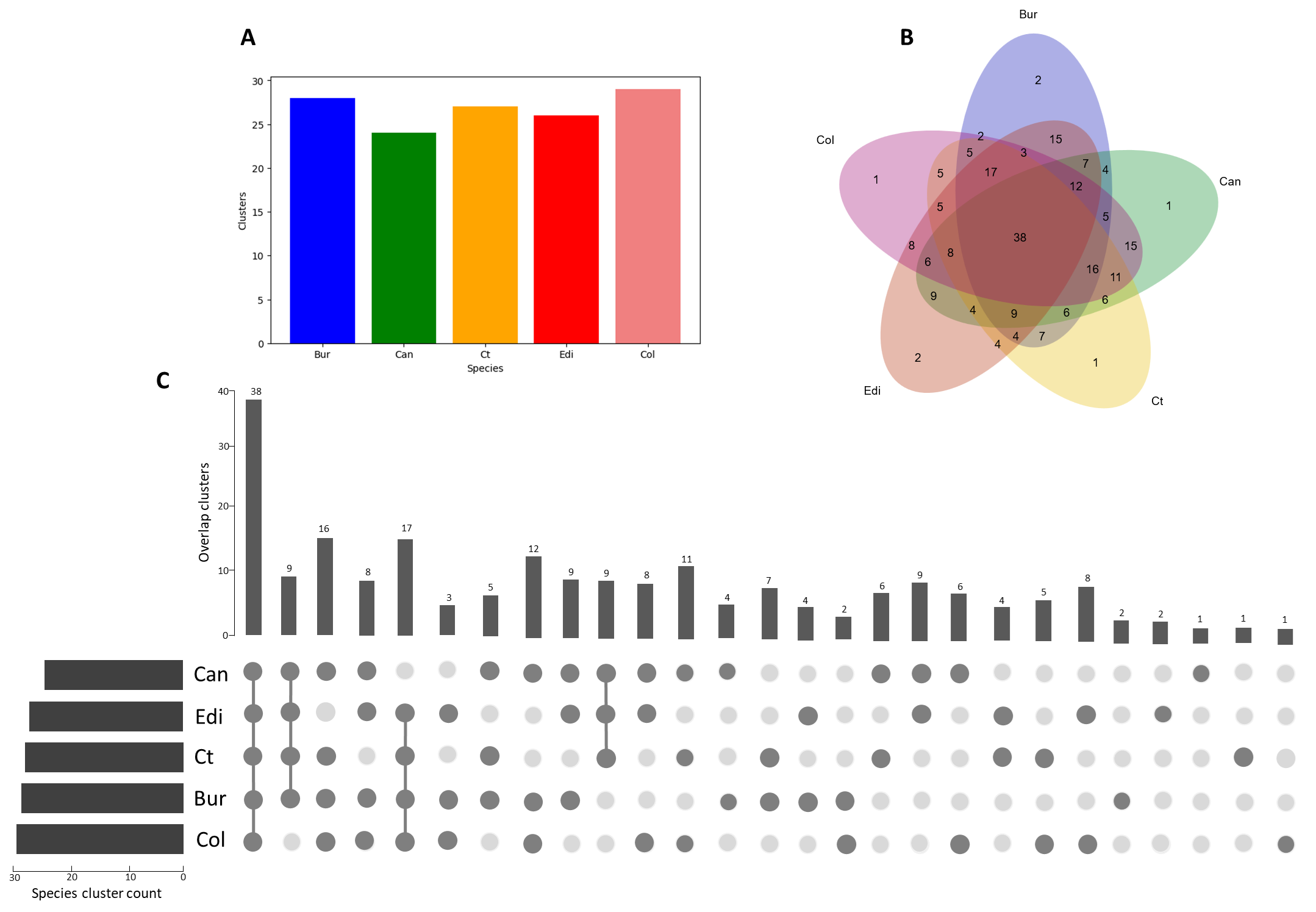


**Supplementary Figure S1:** (**A)** Bar plot represents the orthologous clusters of identified CRK genes from *Bur_0.v7, Can_0.v7, Ct_1.v7, Edi_0.v7, Col_0* *Arabidopsis* ecotypes genomes, respectively, (**B)** Venn diagram represents the pangenes: the core, dispensable, and unique clusters of the identified CRK members, and (**C)** Upset plot represents the overlapping and unique clusters among five *Arabidopsis* ecotypes genomes.


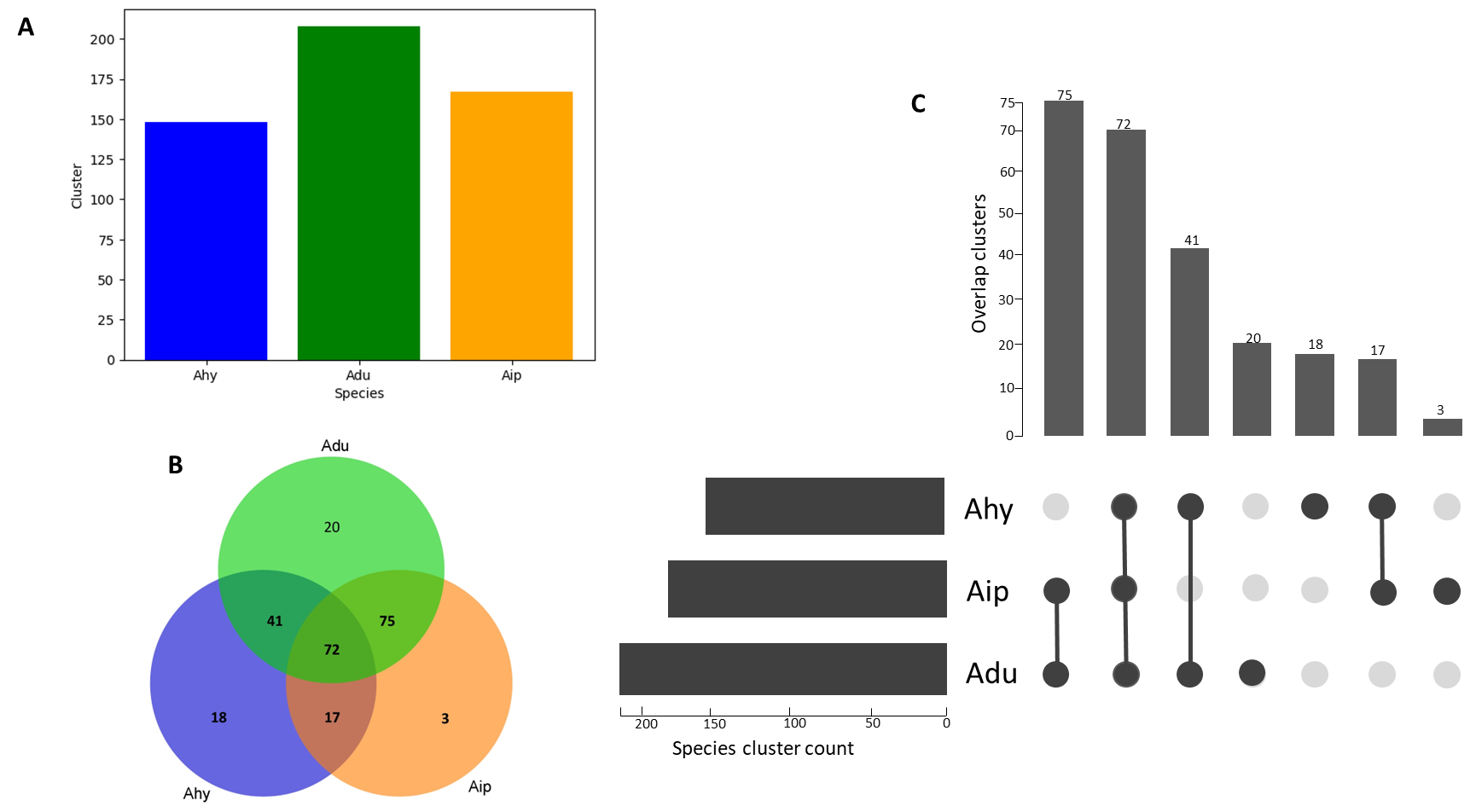


**Supplementary Figure S2:** (**A)** Bar plot represents the orthologous clusters of identified CRK genes from *A. hypogaea*, *A. duranensis*, and, *A. ipaensis*, (**B)** Venn diagram represents the pangenes: the core, dispensable, and unique clusters of the identified members, and (**C)** Upset plot represents the overlapping and unique clusters of overlapping *Arachis CRK* gene family members.

**
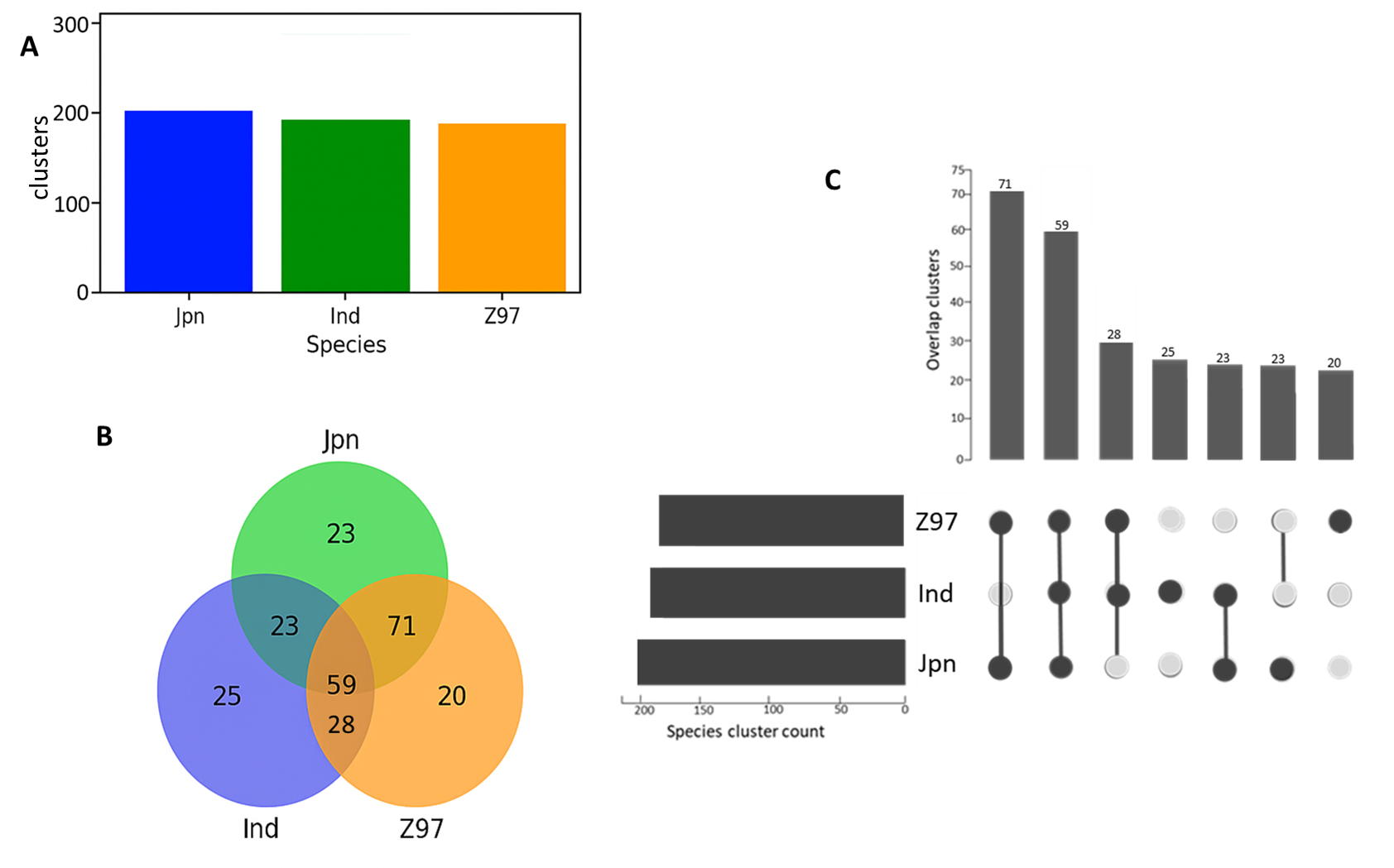
**

**Supplementary Figure S3:** (**A)** Bar plot represents the orthologous clusters of identified CRK genes from three rice cultivars, *O. sativa Japonica Group* Nipponbare (Jpn), O*. sativa* indica 93–11 (Ind), and *O. sativa* Zhenshan 97 (Z97); (**B)** Venn diagram represents the pangenes: the core, dispensable, and unique clusters of the identified members; and (**C)** Upset plot represents the overlapping and unique clusters of overlapping rice *CRK* gene family members.


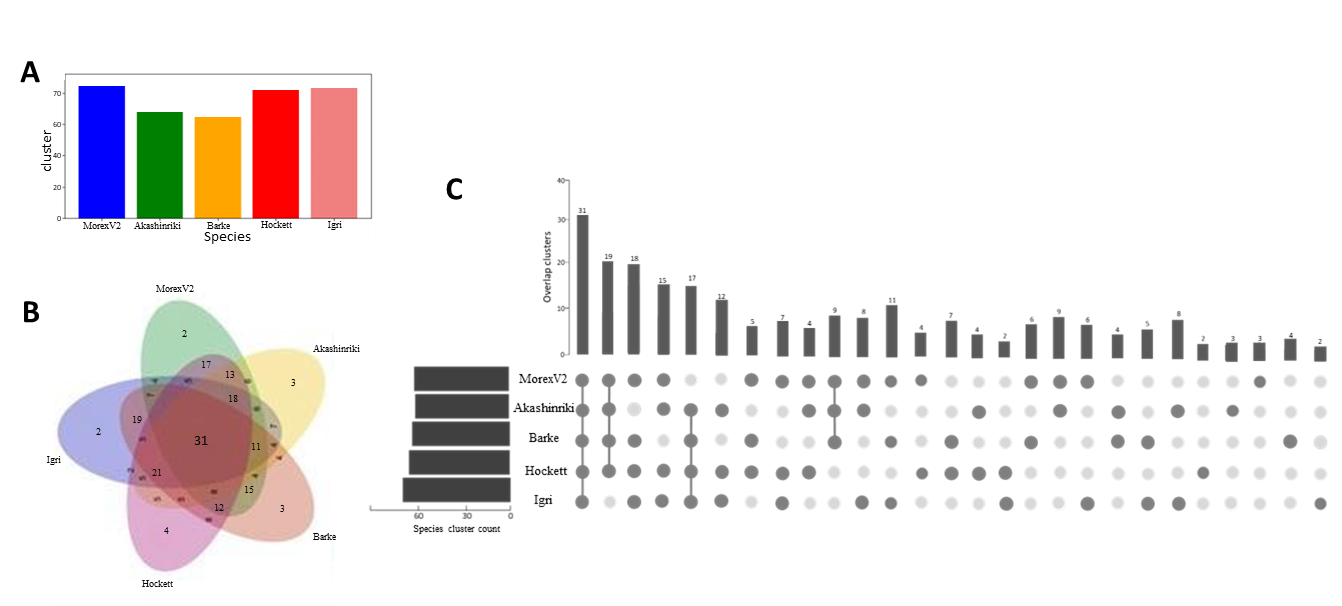


**Supplementary Figure S4:** (**A)** Bar plot represents the orthologous clusters of identified CRK genes from MorexV2, Akashinriki, Barke, Hockett, Igri genomes, respectively, (**B)** Venn diagram represents the pangenes: the core, dispensable, and unique clusters of the identified CRK members, and (**C)** Upset plot represents the overlapping and unique clusters among five Barley genomes.

**Supplementary Table 2:** Comparison of PangenePro with similar tools shows the differences or similarities in approaches.

| **Pipeline/Tool/Database** | **Objective** | **Approach/Method** | **Comparison** |
| --- | --- | --- | --- |
| PangenePro | Gene family identification across multiple genomes, pangene identification | Alignment (BLAST), Domain profiling, ortholog clustering, pangenes-based classification | - |
| GET_PANGENES (Moreira et al., 2023) | Pangene detection via whole-genome alignment and phylogeny-based orthologies | Whole-genome alignment (WGA) + collinear gene model clustering by confirming presence-absence variation | Synteny-based, involves whole genome comparison, no domain profiling for specific gene family-based identification. |
| Pandagma (Cannon et al., 2024) | Identification of pan-gene sets and gene families at desired evolutionary depths and accommodating whole-genome duplications | Use a combination of homology, synteny, and expected rates of synonymous change in coding sequence | Requires more configuration; lacks full automation for raw input data handling. |
| PLAZA 5.0 (Bel et al., 2022) | Platform for comparative, evolutionary studies of green plant gene families, and functional plant genomics | Predefined orthogroups, limited gene families and plant species, collinearity, enrichment analysis | Extensive resource, but not suitable for user-uploaded genome sets and pangenes-based study. |
| Orthofinder (Emms & Kelly, 2019) | Automated and accurate orthogroup, ortholog, and gene tree inference from proteomes | All-vs-all DIAMOND/BLAST, orthogrouping, rooted species/gene trees, duplication-loss-coalescence model | More phylogeny-focused with rooted gene/species trees; does not classify into pangene sets |
| GENESPACE (Lovell et al., 2022) | Tracks regions of interest and gene copy number variation across (CNV) multiple genomes | Uses synteny and gene CNV tracking across genomes, it focuses on cross-genome structural comparison with evolutionary context | No direct gene family identification and pangene level analysis |
| OrthoMCL (L. Li et al., 2003) | Clustering of orthologs and paralogs across multiple species using sequence similarity | All-vs-all BLAST, MCL clustering | Performs gene family comparison but not initial identification. |
| SPUMONI 2 (Ahmed et al., 2023) | Tool for genomics analyses use large reference sequence collections, like pangenomes or taxonomic databases. | Sequence classification using pangenome minimizer index, r-index over minimizer digest for short/long read classification | Not designed for gene family analysis or ortholog clustering. Limited to classification of long and short reads, no downstream / pangenes level analysis |
| PlantTribes2 (Wafula et al., 2023) | Objective classifications of annotated protein sequences from genomes or transcriptomes for comparative and evolutionary analyses of gene families from any type of organism | Orthogroup assignment, sequence alignment, phylogenetics, using genomics and transcriptomics datasets, accessible through GitHub | Identifies gene families but doesn’t perform pangenes or pangenome-level analyses |
| CropGF (Xu et al., 2023) | Platform for crop gene family mining and further downstream analysis | Database search for gene families and their members using domains, BLAST,  Limited to six crop species | Limited batch input, user-defined genomes are not supported, no pangenome-level analysis or classification |
| PlantGF (J. Li et al., 2022) | A comprehensive analysis and annotation platform for plant gene families encompassing 138 plant species and their gene families | Pre-computed families + online tools (HMMER, MAFFT, BLAST) | Query-focused; no batch or dynamic input analysis, No novel gene family discovery or pangenome-wide analysis/classification |
| PGSB PlantsDB (Spannagl et al., 2016) | A database framework for comparative plant genome research | Based on sequence mining using BLAST | Not automated, limited to a few plant species, no pangene level analysis |
| MGFD (Sheng et al., 2016) | Database of manually curated maize gene families. most gene families are transcription factor families | Similarity search, feature annotation | Maize-specific, limited gene families, not an automated or pan-genome analysis-based approach |

Ahmed, O. Y., Rossi, M., Gagie, T., Boucher, C., & Langmead, B. (2023). SPUMONI 2 : improved classification using a pangenome index of minimizer digests. *Genome Biology*, 1–15. https://doi.org/10.1186/s13059-023-02958-1

Bel, M. Van, Silvestri, F., Weitz, E. M., Kreft, L., Botzki, A., Coppens, F., & Vandepoele, K. (2022). *PLAZA 5 . 0 : extending the scope and power of comparative and functional genomics in plants*. *50*(November 2021), 1468–1474.

Cannon, S. B., Lee, H.-O., Weeks, N. T., & Berendzen, J. (2024). Pandagma: A tool for identifying pan-gene sets and gene families at desired evolutionary depths and accommodating whole genome duplications. *Bioinformatics*, btae526. https://doi.org/10.1093/bioinformatics/btae526

Emms, D. M., & Kelly, S. (2019). OrthoFinder: phylogenetic orthology inference for comparative genomics. *Genome Biology*, *20*, 1–14.

Li, J., Yang, S., Yang, X., Wu, H., Tang, H., & Yang, L. (2022). *PlantGF : an analysis and annotation platform for plant gene families*. *2022*(January), 1–5.

Li, L., Stoeckert, C. J., & Roos, D. S. (2003). OrthoMCL: identification of ortholog groups for eukaryotic genomes. *Genome Research*, *13*(9), 2178–2189.

Lovell, J. T., Sreedasyam, A., Schranz, M. E., Wilson, M., Carlson, J. W., Harkess, A., Emms, D., Goodstein, D. M., & Schmutz, J. (2022). GENESPACE tracks regions of interest and gene copy number variation across multiple genomes. *Elife*, *11*, e78526.

Moreira, B. C., Saraf, S., Naamati, G., Casas, A. M., Amberkar, S. S., Flicek, P., Jones, A. R., & Dyer, S. (2023). GET _ PANGENES : calling pangenes from plant genome alignments confirms presence ‑ absence variation. *Genome Biology*, 1–18. https://doi.org/10.1186/s13059-023-03071-z

Sheng, L., Jiang, H., Yan, H., Li, X., & Lin, Y. (2016). *Database update MGFD : the maize gene families database*. 1–5. https://doi.org/10.1093/database/baw004

Spannagl, M., Nussbaumer, T., Bader, K. C., Martis, M. M., Seidel, M., Kugler, K. G., Gundlach, H., Mayer, K. F. X., & Link, S.-. (2016). *PGSB PlantsDB : updates to the database framework for comparative plant genome research*. *44*(November 2015), 1141–1147. https://doi.org/10.1093/nar/gkv1130

Wafula, E. K., Zhang, H., Von Kuster, G., Leebens-Mack, J. H., Honaas, L. A., & dePamphilis, C. W. (2023). PlantTribes2: Tools for comparative gene family analysis in plant genomics. *Frontiers in Plant Science*, *13*. https://www.frontiersin.org/journals/plant-science/articles/10.3389/fpls.2022.1011199

Xu, J., Zhu, C., Su, M., Li, S., Chao, H., & Chen, M. (2023). *CropGF : a comprehensive visual platform for crop gene family mining and analysis*. *00*(00), 1–7. https://doi.org/https://doi.org/10.1093/database/baad051
